# Molecular Mechanism of Global Genome Nucleotide Excision Repair

**Published:** 2014

**Authors:** I.O. Petruseva, A. N. Evdokimov, O. I. Lavrik

**Affiliations:** Institute of Chemical Biology and Fundamental Medicine, Siberian Branch of the Russian Academy of Sciences, prosp. Akad. Lavrentyeva, 8, 630090, Novosibirsk, Russia; Altai State University, Ministry of Education and Science of the Russian Federation, prosp. Lenina, 61, 656049, Barnaul, Russia; Novosibirsk State University, Ministry of Education and Science of the Russian Federation, Pirogova Str., 2, 630090, Novosibirsk, Russia

**Keywords:** nucleotide excision repair, repair factors, molecular mechanisms of damage recognition and elimination

## Abstract

Nucleotide excision repair (NER) is a multistep process that recognizes and eliminates a wide spectrum of damage causing significant distortions in the DNA structure, such as UV-induced damage and bulky chemical adducts. The consequences of defective NER are apparent in the clinical symptoms of individuals affected by three disorders associated with reduced NER capacities: xeroderma pigmentosum (XP), Cockayne syndrome (CS), and trichothiodystrophy (TTD). These disorders have in common increased sensitivity to UV irradiation, greatly elevated cancer incidence (XP), and multi-system immunological and neurological disorders. The eucaryotic NER system eliminates DNA damage by the excision of 24–32 nt single-strand oligonucleotides from a damaged strand, followed by restoration of an intact double helix by DNA repair synthesis and DNA ligation. About 30 core polypeptides are involved in the entire repair process. NER consists of two pathways distinct in initial damage sensor proteins: transcription-coupled repair (TC-NER) and global genome repair (GG-NER). The article reviews current knowledge on the molecular mechanisms underlying damage recognition and its elimination from mammalian DNA.

## INTRODUCTION

**Fig. 1:**
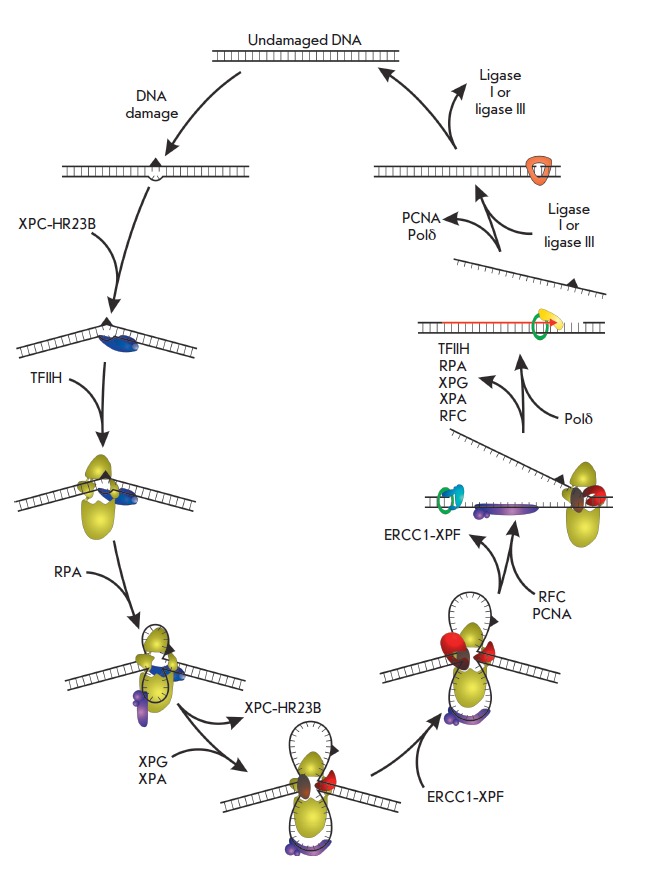
Scheme of global genome excision repair for nucleotides

Nucleotide excision repair (NER ) is one of the principal ways in which cells are protected against various, structurally and chemically different, DNA lesions. The most common lesions are bulky covalent adducts, which are formed by nitrogenous bases affected by UV light, ionizing irradiation, electrophilic chemical mutagens, some drugs, and chemically active endogenous metabolites, including reactive oxygen and nitrogen species [1]. In higher eukaryotic cells, NER excises 24-32-nt DNA fragments containing a damaged link with extreme accuracy. Reparative synthesis using an undamaged strand as a template, followed by ligation of the singlestrand break that emerged as a result of the damage, is the final stage of DNA repair. Currently available information on the main genes inactivated in NER -defective cells and on the protein factors and enzymes encoded by these genes indicates that the process involves the coordinated action of approximately 30 proteins that successively form complexes with variable compositions on the DNA [1-3]. NER consists of two pathways distinct in terms of initial damage recognition. Global genome nucleotide excision repair (GG-NER ) detects and eliminates bulky damages in the entire genome, including the untranscribed regions and silent chromatin, while transcription-coupled nucleotide excision repair (TC -NER ) operates when damage to a transcribed DNA strand limits transcription activity. TC -NER is activated by the stopping of RN A polymerase II at the damaged sites of a transcribed strand, while GG-NER is controlled by XPC, a specialized protein factor that reveals the damage. A schematic GG-NER process is presented in *Fig. 1*; information on the main proteins participating in the process is presented in Table.

Distortions in NER activity can result in UV-sensitive and high-carcinogenic pathologies, xeroderma pigmentosum (XP), the Cockayne syndrome (CS), and trichothiodystrophy (TT D), as well as some neurodegenerative manifestations [4-6].

Xeroderma pigmentosum has provided the names of some of the genes that cause (when being mutated or distorted) the symptoms associated with the disease and the proteins coded by these genes (XPA–XPE factors). XP is a syndrome characterized by photosensitivity, skin atrophy, hyperpigmentation and a high rate of sunlight-induced skin cancer. The risk of internal tumors in XP patients is at least 1,000-fold higher [6, 7]. Moreover, the disease is often associated with neurologic disorders. Various XP symptoms, typical of seniors, indicate premature aging caused by the accumulation of non-repaired bulky DNA damage, including several oxidative ones [8-10].

**List of used antibodies:** 

Factor	Subunit	Gene	Weight, cDNA / (size, a.a.r.)	Function within NER	Interaction with other factors
XPC	HR23B	hhr23b	43 / (409)	Recognition of a distorted DNA structure	TFIIH XPA DDB
	XPC	xpc	125 / (940)		
	CEN 2	cen2	20 / (172)		
DDB	DDB1	ddb1	127 / (1140)	Recognition of damage, interaction with chromatin	XPC RPA
	DDB1	ddb2	48 / (428)		
XPA	XPA	*xpa*	31 / (273)	Structural function, binding to a damaged strand	XPA RPA TFIIH ERCC 1
RPA	RPA70	rpa1	68 / (616)	Binding to single-stranded DNA	XPA XPG PCN A/RFC
	RPA32	rpa2	30 / (270)		
	RPA14	rpa3	14 / (121)		
TFIIH	XPB	xpb	89 / (782)	ATPase, minor helicase activity 3'→5'- DNA-helicase	XPA XPC XPF XPG
	XPD	xpd	87 / (760)	ATP-dependent 5'→3'-DNA-helicase; testing of modification presence	
	p62	gtf2h1	62 / (548)	Core subunit, stimulates XPB	
	p44	gtf2h2	gtf2h2 44 / (395)	Core subunit, stimulates XPD	
	p34	gtf2h3	34 / (308)	DNA binding	
	p52	gtf2h4	52 / (462)	Regulatory subunit for ATPase activity of XPB functioning in TFIIH complex	
	p8	gtf2h5 (ttda)	8 / (71)	Interaction with P52, stimulation of ATPase activity of XPB	
	Mat1	mnat1	36 / (309)	Member of the CAK complex	
	Cdk7	cdk7	39 / (346)	Phosphorylates RN A-polymerase II and other substrates	
	Cyclin	ccnh	38 / (323)	Regulation of cell cycle	
XPF	ERCC1	ercc1	33 / (297)	Endonuclease, catalyzes formation of single-strand break in DNA on the 5’ side of the damage	XPA TFIIH
	XPF	xpf	103 / (905)		
XPG	XPG	xpg	133 / (1186)	Endonuclease, catalyzes formation of single-strand break in DNA on the 3’ side of the damage	TFIIH RPA PCN A
RFC	RFC1	rfc1	128 / (1148)	ATP-dependent connection of PCN A	PCN A RPA
	RFC2	rfc2	39 / (354)		
	RFC3	rfc3	41 / (356)		
	RFC4	rfc4	40 / (363)		
	RFC5	rfc5	38 / (340)		
PCNA	PCNA	pcna	3X37 / (3X261)	Factor ensuring processivity of DNA polymerases	RFC XPG Polδ
Polδ	p125	p125	124 / (1107)	DNA polymerase	PCNA
	p66	p66	51 / (466)		
	p50	p50	51 / (469)		
	p12	p12	12 / (107)		
Polε	p261	p261	261 / (2286)	DNA polymerase	PCNA
	p59	p59	60 / (527)		
	p17	p17	17 / (147)		
	p12	p12	12 / (117)		
Ligase I	Ligase I	ligI	102 / (919)	Ligation of a single-strand break	
Ligase III	Ligase III	ligIII	103 / (862)		

## DAMAGE RECOGNITION

Damage recognition is the crucial step of NER initiation; it determines the rate of DNA repair [1, 2, 11]. A distorted regular structure of double-stranded DNA (dsDNA) and alteration of its stability are common signs conditioning the initial recognition of damage by the repair systems. Chemical modifications of nitrogenous bases are the elements most often eliminated by the base excision repair (BER ) system. Pyrimidine photodimers, platinum adducts, protein-DNA cross-links, modifications caused by DNA interaction with active derivatives of benzo[a]pyrene, benzo[c]anthracene, acetylaminofluorene, along with other bulky adducts, which cause more substantial distortions in the regular structure of double-stranded DNA than BER repairable damages, are the most typical NER substrates [12]. However, most of NER substrates cannot cause as dramatic structural and thermodynamic alterations of dsDNA as double-strand breaks and interstrand crosslinks. Therefore, the detection of these damages is particularly challenging for a cell, which can be solved only through highly sensitive recognition. In contrast to BER , where a damaged base is simultaneously recognized and eliminated by a single specialized glycosylase, spezialized groups of proteins are responsible in NER for each of the processes. In eukaryotic NER universal sensor proteins perform the initial recognition of the total range of bulky damages. In the case of TC -NER , it is transcribing RN A polymerase II stopped by damage; in GG-NER , these are complexes of the XPC factor and DDB1-DDB2 heterodimer (XPE factor) enhancing the repair of UV damage [1, 2]. In general, NER recognition of damage is a multistep process involving several proteins that form near damaged complexes of variable compositions. The process is completed by the formation of a preincision complex ready to eliminate a damaged DNA fragment by specialized NER endonucleases [1, 2].

Complementary interaction of nitrogen bases is the main factor ensuring the stability of a regular helical structure of double-stranded DNA. Bulky damage causes distortion in base-pairing and occurrence of a single-stranded character in a dsDNA molecule. Undamaged DNA is not a static molecule, either. DNA strands are in continuous heat motion, causing small, rapid alterations of the distances separating the complementary bases. However, these pico- and nanosecond fluctuations existing in the undamaged DNA may be too short in order to be recognized by repair factors. Molecular modeling shows that introduction of bulky damage into DNA can give rise to more considerable and long-lived “openings” in the double helix [13, 14]. For example, such fluctuations in the DNA structure occur near the cyclobutane pyrimidine dimer 25-fold as often as those in an undamaged duplex. Moreover, the fluctuation’s amplitude increases crucially due to a disturbed interaction between the complementary DNA strands. The dynamic changes that follow nucleobase damage mostly cause fluctuations in an undamaged strand fragment that is complementary to the one containing the lesion, while the damaged DNA fragment is less flexible [15, 16]. These fluctuations may mediate the recruitment of the repair factors that recognize damage at the initial stages. Results of experiments (in particular, the analysis of specific excision efficiency using model DNAs with various structures, which became the grounds for formulating the concept of the bipartite recognition process in NER ) point to the important role of the intact DNA strand in the recognition process [15, 16]. NER proteins from a cellular extract can initiate the repair process only when the model DNA is characterized both by a chemical modification and distortions in the secondary structure. Thus, a fragment containing the C4’-pivaloyl adduct of deoxyribose, a bulky but not distorting structure of the regular DNA duplex, was excised only when it was located in an artificialy short site of a pairing distortion. The sites of modification-free uncoupled bases cannot act as substrates for specific excision; neither can structures containing a chemical modification opposite to the loop formed by an unmodified strand [16].

Numerous studies have been devoted to the search for the proteins responsible for initial damage recognition and recruitment of the following NER factors. Although a number of facts point to the key role of XPC in the initiation of NER [17-19], the results of the evaluation of their affinity to damaged DNA and analysis of the specificity to a damaged substrate have provided opportunity to consider the XPA factor and its complexes with RPA and XPC as a damage sensor [20-23]. Confocal microscopy using fluorescent proteins has shown that XPC can be immobilized near UV damages in the absence of XPA (XPA-deficient cells), while in XPC-deficient cells, XPA is not bound to the damaged DNA sites [3, 18]. The results of biochemical studies have shown that XPC is required for the recruitment of other factors into the GG-NER process [17, 19, and 24]. Various approaches that have included visualization methods allowing to track fluorescent protein movements within chromatin in a living cell have been applied to clarify the mechanism whereby XPC recognizes the damage against a background of an excess of intact DNA. FRAP/FLIP (fluorescence recovery after photobleaching/ fluorescence loss in photobleaching). It was shown that the dynamics of the movement and intranuclear localization mode of GFP-XPC differ from the dynamics and other NER factors localization (GFP-XPA, TFIIH-GFP). XPC permanently scans the genome DNA in search of damage. The scanning mode is associationdissociation with the formation of a plethora of shortlived complexes. More stable XPC-DNA complexes are formed when XPC collides with damaged sites, following which the recruitment of other NER factors to the damaged site occurs. In addition, XPC is permanently exported from the nucleus and imported back. Such XPC exchange in the absence of damage maintains the stationary level of its nuclear concentration, preventing redundant DNA probing that may interfere with other processes of nucleic metabolism. Under any effects on cells resulting in DNA damage, the rate of XPC transport to the cell decreases and XPC accumulates in the nucleus, which facilitates the rapid response of the repair system to genotoxic affection. This effect is maximally pronounced when NER -repaired damage arises. The XPC nucleus-cytoplasmic exchange is delayed for 6–8 h, exceeding markedly the time of the XPC presence in NER complexes. Some authors [25] regard the slow repair of some types of UV damage as the reason behind such a prolonged XPC exchange stop. XPC needs heterodimer UV-DDB as a partner protein to recognize UV damage efficiently [26-29].

The molecular basis of XPC-DNA interaction is now being actively examined. A detailed understanding of the mechanism of initial recognition of a DNA substrate by a sensor protein conditions the understanding of the interplay between the damaged structure and its rate of excision from the DNA, as well as the way by which factor XPC discriminates damaged nucleotides against a background of a substantial excess of undamaged DNA. The X-ray diffraction analysis of Rad4, a yeast ortholog of XPC, provided considerable progress in the study of the structure of a sensor protein-damaged DNA complex. The analysis of the structure of the crystallized complex of truncated Rad4 (a.a.r. 123–632) + Rad23 protein + heteroduplex containing the cyclobutane- pyrimidine dimer has shown that a large (transglutaminase, TGD) Rad4 domain with one β-hairpin from domain 1 (BHD1) forms a C-shaped structure by coming into contact with 11 nucleotides of the undamaged dsDNA on the 3’ side of the damage. Another portion of Rad4 is composed of the hairpin domains BHD2 and BHD3 that mainly form van der Waals contacts with the DNA substrate near the damage site. The long β-hairpin emerging from BHD3 is inserted into the double helix, causing the DNA backbone to bend. As a result, both the cross-linked pyrimidines and the opposite bases of the undamaged strand are displaced from the helix. The protein does not come into contact with the damage directly, interacting with two adjacent bases and two bases opposite CPDs. Each adjasent undamaged base is clamped between residues of aromatic amino acids from the BHD2/BHD3 motif [30]. This is a typical mode of interaction between the OB-subdomain (a structural unit present in proteins with increased affinity to single-stranded DNA) and ssDNA [31]. The image of Rad4 matches well our understanding of the way XPC interacts with a damaged DNA based on the data on this protein structure and the results of biochemical examinations. The analysis by atomic force microscopy has shown that XPC binding results in the bending of the DNA-duplex backbone and formation of a ~140– 130° angle [32]. As shown by permanganate footprinting the emerging bend of the helix axis of damaged DNA is followed by partial melting of the duplex (by approximately 4–7 nucleotides) [33]. The similarity between the schemes of location of the RAD4 and XPC factors on damaged DNA is confirmed by the results of photo-induced cross-linking of these proteins with DNA containing a bulky modification [34]. This pattern of XPC-DNA interaction, the strategy of indirect check for the presence of structural lesions, resulting in an increased level of fluctuations in the undamaged strand, underlies the incredibly wide substrate specificity of the GG-NER pathway. The transglutaminase domain and a domain structurally similar to the OB-subdomain of factor RPA were found in human XPC; the domains interact with ssDNA with the use of an aromatic damage sensor, a pair of aminoacid residues, Trp690, and Phe733 [35-37].

FRAP experiments using XPC forms truncated both at the N- and C-ends have revealed the XPC fragment mainly responsible for the recognition of damaged DNA. The fragment comprising, in fact, only 15% of the full-size XPC (a minimal sensor) appears to be capable of UV damage recognition in live cells. The minimal sensor fragment prefers heteroduplexes and single-stranded oligonucleotides; it recognizes damage due to its affinity to the regions with distorted hydrogen bonds. The fragment consists of BHD1, BHD2, and a short (25 amino acid residues) motif separating the BHD2 and BHD3 domains and is folded to form a structure known as a β-turn. Specific features of the β-turn determine the operational efficiency of a minimum damage sensor [38, 39]. This short polypeptide fragment can either be attracted to or repulsed by DNA; due to this feature, an XPC is capable of dynamic interaction with DNA within the genome. Damage recognition is facilitated in this case, providing the DNAscanning molecules of the sensor protein with sufficient mobility. The truncated C-terminal XPC containing a β-turn keeps some residual repair activity found using the cell reactivation method. A photobleaching assay of protein motion dynamics proves increased XPC mobility in the nuclei of living cells [24]. The same approach demonstrates that rapid post-UV-immobilization of XPC occurs only in the nuclei of cells containing XPC mutants with an intact β-turn. Especially remarcable is the fact that the polypeptide fragment including BHD1 and BHD2 also acts as a minimal sensor only if an intact β-turn is presented. Biochemical experiments show that the XPC nuclear mobility determined by the structural element results from the repulsion of a protein molecule from an undamaged dsDNA. Finally, the dynamic role of a β-turn within a full-size XPC was confirmed by the results of site-directed mutagenesis when glutamic acid was replaced with lysine. This charge inversion was supposed to reduce the strength of electrostatic repulsion between a negatively charged lateral chain of a protein and the phosphates within the DNA backbone. As was assumed, the charge inversion increased the affinity of mutant XPC molecules to undamaged DNA, reducing their mobility within the nucleus and decreasing the activity of the GG-NER pathway. Thus, the β-turn plays a crucial role in the regulation of the dynamics of XPC–normal DNA duplex interaction. This subdomain, due to its ability to repulse DNA, facilitates damage recognition, providing sufficient mobility to the XPC molecules that search for genome damage [24, 35-38]. When XPC binds to the abnormally oscillating region of a native strand in a way that excludes direct contacts with the damage, the nucleoprotein intermediates formed upon initial screening can be converted into a strong recognition complex [29, 36-39].

Within a cell, XPC exists as the heterotrimeric complex XPC-HR23B-Cen2 [1, 2, 18]. HR23B stabilizes the complex, protects it against proteasome degradation, and stimulates the DNA-binding activity of XPC. The recombinant heterodimer XPC-HR23B is a stable complex that interacts *in vitro *with damage of various types and is widely used for the NER reaction in a reconstituted system [18, 40, 41]. The interplay between XPC-HR23B and damaged DNA was analyzed using affinity modification. DNA duplexes of various structures containing bulky modifications, including photoactive fluorochloroazide pyridyl damage, were used as probes. Some model duplexes contained analogs of undamaged strands created with the use of photo reagents with a zero linker length: nucleotide links with 4-thio- and 5-iodo-modified bases [34, 42-44]; some duplexes included a platinum adduct [45]. A large XPC subunit was the only modification target in all cases. The second high-molecular weight nucleoprotein adduct with a lower electrophoretic activity appeared as a result of photo-induced cross-linking with other amino acid residues of the DNA-binding XPC subunit [44]. Moreover, the product of XPC-HR23B proteinprotein cross-links emerging after hard (254 nm) and long-term (60 min) UV irradiation and revealed by Western blotting does not contain a radioactive label and can be formed independently of the presence of a DNA probe [45]. The HR23B subunit of the complex does not come into contact with DNA directly; this was shown by the absence of products of its photo-induced cross-linking with analogues of a damaged DNA. Quite recently, confocal microscopy showed that HR23B, in contrast to XPC, is not immobilized on the damaged DNA of a cell and is released from the complex after XPC binding [46].

The roles played by centrin-2 in the XPC complex have not been completely clarified, though the presence of the protein is known to increase the stability, control affinity/selectivity of DNA binding by the XPC-HR23B dimer. Also Cen2 interaction with the Cend fragment of XPC can regulate the recruitment of TFIIH [35].

Binding of TFIIH to the nucleoprotein complex formed by damaged DNA and XPC triggers the verification of the damaged DNA as a NER substrate; that is, the presence of a bulky chemical modification in the discovered XPC DNA site with a distorted regular structure.

## DAMAGE VERIFICATION AND ASSEMBLY OF THE DAMAGED FRAGMENT OF A COMPLEX READY FOR EXCISION

**Fig. 2:**
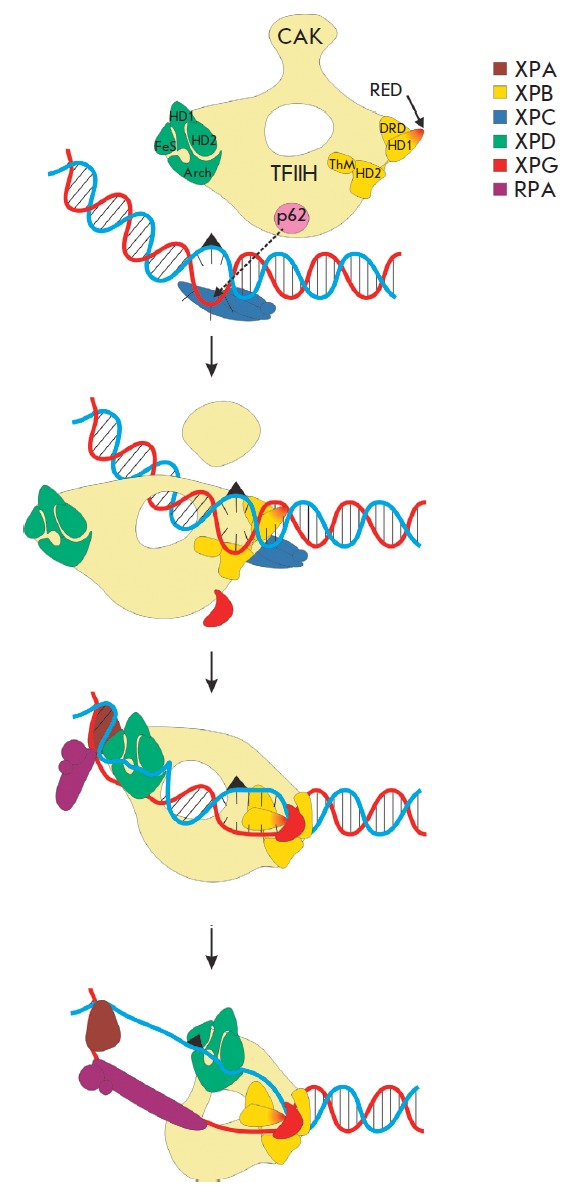
Scheme of the two-step process of damage recognition

The TFIIH factor is a multisubunit complex composed of two helicases, XPB and XPD; enzymatic activityfree proteins, p62, p52, p44, p34 and p8; and the complex of CDK-activating kinase, CAK (cyclin H, Cdk7, and Mat1). In a 3D model of human TFIIH, established according to the results of an electron microscopic analysis, the core proteins form a slightly elongated ring-shaped structure (16 × 12.5 × 7.5 nm) with a hole of a diameter sufficient to enclose a double-stranded DNA helix (2.6–3.4 nm) [47]. A structure formed by core proteins via XPD contacts with the CAK subcomplex, forming a bulge on the external side of the ring. The smallest p8 subunit (TT DA) is also included into the core composition. XPC-dependent recruitment of TFIIH to the damage is mainly controlled by direct contact of XPC with the XPB and p62 subunit (*Fig. 2*). The TFIIH annular structure encompasses the dsDNA on the 5’ side of the damage, releasing a kinase subcomplex. Uncoiling of a DNA double helix around the damage catalyzed by two specialized helicases, XPB (3'→5') and XPD (5'→3'), is the most obvious result of TFIIH binding. It is followed by the formation of an approximately 27 nucleotide-long (22 nucleotides on the 5’ side of the damage and 5 nucleotides – on the 3’ side) asymmetrical region of separated strands. This stage requires the energy of ATP hydrolysis [48-51]. The mechanism of formation of single-stranded DNA regions around the damage and checking for modification presence become clearer thanks to the data on the structure of the XPB and XPD factors, obtained in the study of the crystal structure of protein analogues of archaea [52-54], and the analysis of the structure of the C-terminal fragment of human XPB [53]. Analysis of the structure of *Archaeoglobus fulgidus *XPB crystals showed that the protein contains two helicase domains, HD1 and HD2, including seven helicase motifs. Two new structural motifs, RE D in HD1, consisting of three charged amino acid residues – Arg, Glu, and Asp – and a thumb-like motif (ThM) in HD2, similar to that found in T7-DNA polymerase. Each analog of the human XPD from three archaeal species (*Thermoplasma acidophilum, Sulfolobus tokodaii, *and *S. acidocaldarius*) contains four domains, including HD1, HD2, Archdomain, and the unique 4FeS-domain comprising the Fe-4S-claster, which was found for the first time in the helicase structure [54-56]. The details of XPD–DNA interaction and structure of the established complexes have been actively examined using the models of recombinant archaeal helicases. The established model of XPD–DNA interaction supposes that ssDNA is bound in a groove between the Arch and HD2 domains and passes through a hole (a pore) in a globule with a diameter sufficient for free helicase motion along the DNA. Bulky adducts repaired by the NER pathway might block XPD translocation along the ssDNA located in such a way. This idea is in accordance with earlier data on the inhibiting activity of a yeast XPD analog, rad3 helicase, as it interacts with a bulky damage [57]. An XPD analog from *Ferroplasma acidarmanus*, which acts in the form of a monomer but is structurally similar to the human protein, helicase was shown to be stopped by damage in the strand along which it translocates in the 5’→3’ direction. In contrast to the inhibited helicase activity, the ATPase activity of a damagebound XPD is preserved and even increases. Moreover, when a complementary 3’→5’ strand contains CPD, the enzyme dissociates from the substrate [58]. The data on the crystal structures of archaeal XPD homologs supports the idea that the presence of a modification in DNA is finally verified when a base binds to the pocket located on the XPD surface. The pocket is located near the tunnel within the protein structure used to thread a DNA strand [54, 56, 58]. Examination of the interactions between mutant human XPD proteins and DNA containing UV damage definitely confirmed the idea that the XPD subunit of TFIIH checks for the presence of damage. The mutations were inserted into the protein region located in the site of the DNA-binding channel-pore transition (a.a.r. Y192A and R196E). The amino acid residues directly involved in the helicase and ATPase activity were unaffected. The mutant proteins retained their ability to uncoil DNA but could not distinguish between damaged and undamaged DNAs; when these residues were replaced, the XPD ability to form protein complexes (stable recognition intermediates) decreased. Thus, it was demonstrated that these amino acid residues are part of a polypeptide fragment forming a sensor pocket of human XPD. The pocket location coincides with that in its archaeal homolog from* T. acidophilum *[59]. In contrast to XPD, the XPB factor moving along the DNA in 3'→5' is more likely to exhibit entraining DNA, brings together the helicase domains 1 and 2 connected by a flexible unstructured fragment acting as a hinge, and forms a site of ATP binding. Composed of charged amino acids the RE D motif of XPB is subsequently inserted between the dsDNA strands and untwists it by approximately 5 nucleotides in the 3’→5’ direction. A preliminary fixation of TFIIH on DNA occurs. A TFIIH ring is inclined with respect to the axis of the DNA helix. XPD acquires the possibility to come into contact with the site of the damaged strand (~22 nucleotides towards the 5’ direction of the damage) and unwinds DNA in the 5'→3' direction when moving along the strand due to the ATP hydrolysis energy and forming an asymmetric bubble. XPD stopes as it encounters a damage site. XPD, together with TFIIH, becomes immobilized on DNA; this situation is typical of bulky modifications [50, 60, ] 60].

After the status of damaged DNA as a NER substrate is confirmed by the emergence of long-lived TFIIHincluding an open nucleoprotein structure the next step of repair starts. A more stable and extended preincision complex is formed; the RPA and XPA factors join the complex. The interactions of RPA and XPA with the TFIIH subunits coordinate the involvement of these proteins in the complex.

RPA is a three-subunit protein factor with very high affinity to single-stranded DNA that participates in many processes of DNA metabolism and is presented in a cell by a large copy number [62]. RPA is required to form the preincision complex and during the following excision of the damaged DNA fragment [1]. Five DNA-binding domains located in the p70 and p32 subunits of RPA have different affinities for substrate, so RPA can form with ssDNA complexes of different architecture and stability. These domains interact with DNA in a polar manner (in 5'→3' direction) [63, 64]. In the preincision complex, RPA occupies approximately 30 nucleotides of the undamaged strand opposite to the damage-containing site, thus protecting DNA from illegitimate degradation and facilitating accurate positioning of XPG and ERCC 1-XPF endonucleases.

XPA, similar to XPC, possesses increased affinity for DNA with a specific secondary structure (in particular, to helix kinks induced by a bulky damage): thus XPA (or its complex with RPA) was considered as a candidate damage sensor or a protein controlling the presence of a modification [1, 65, 66]. However, in contrast to XPC, the XPA factor preferably interacts with a damaged strand and has a much lower affinity for the DNA analogs of NER substrates and intermediates [65, 66]. A small XPA functioning in a cell in monomeric form has a rather complex domain structure. Analysis of the NMR spectra of the DNA-binding XPA fragment formed by the amino acid residues 98–219 revealed a positively charged groove consisting of approximately 60 a.a.r. on the protein surface near the C-end of the DNA-binding domain. The geometric parameters of the groove allow it to bind both to single- and doublestranded DNAs [67, 68]. A zinc finger containing an acid subdomain (a.a.r. 105–129) and a C-end subdomain (a.a.r. 138–209) can be distinguished in the structure of the DNA-binding fragment of XPA. The zinc finger motif does not participate in the DNA binding; it is required for interaction with RPA [67]. The domains of specific XPA interaction with a number of core NER polypeptides, RPA70, RPA32 (N-terminal and central XPA fragments), ERCC 1 (a short region adjacent to the XPA N-terminal fragment), and TFIIH (the XPA Cterminal fragment) were identified using site-directed mutagenesis. XPA-RPA interaction promotes a more efficient binding of both factors to DNA [65, 66, 69], while interaction with a complex formed on the DNA opened around a lesion promotes high selectivity. These XPA properties are the results of structural features allowing for easy changes in conformation and providing efficient interaction with the damaged DNA during the formation of the preincision complex. XPA is currently regarded as a sensor of an anomalous electrostatic potential occurring at the kinks of the negatively charged sugar phosphate DNA backbone. The amino acid residues crucial for efficient XPA functioning were determined by studying the interplay between a series of mutant XPA forms and modified DNAs through gel retardation and photo-induced cross-linking to DNA containing an aryl azide modification [70]. A region of damaged DNA strand that is in contact with XPA was identified using affinity modification. The result of the experiments with a series of probes containing photoactive 5-J-dU and damage-mimicking bulky modification based on fluorescein in various mutual locations shows that most XPA-DNA contacts are located near the ssDNA/ dsDNA junction on the 5’ side of the damage [69]. The ability of XPA to specifically interact with DNA, as well as with many NER proteins (RPA, ERCC 1-XPF, TFIIH, XPC), determines its considerable structural and functional role in the assembly of a complex ready for double incision [71-74].

## ELIMINATION OF A DAMAGED FRAGMENT FROM the DNA

**Fig. 3:**
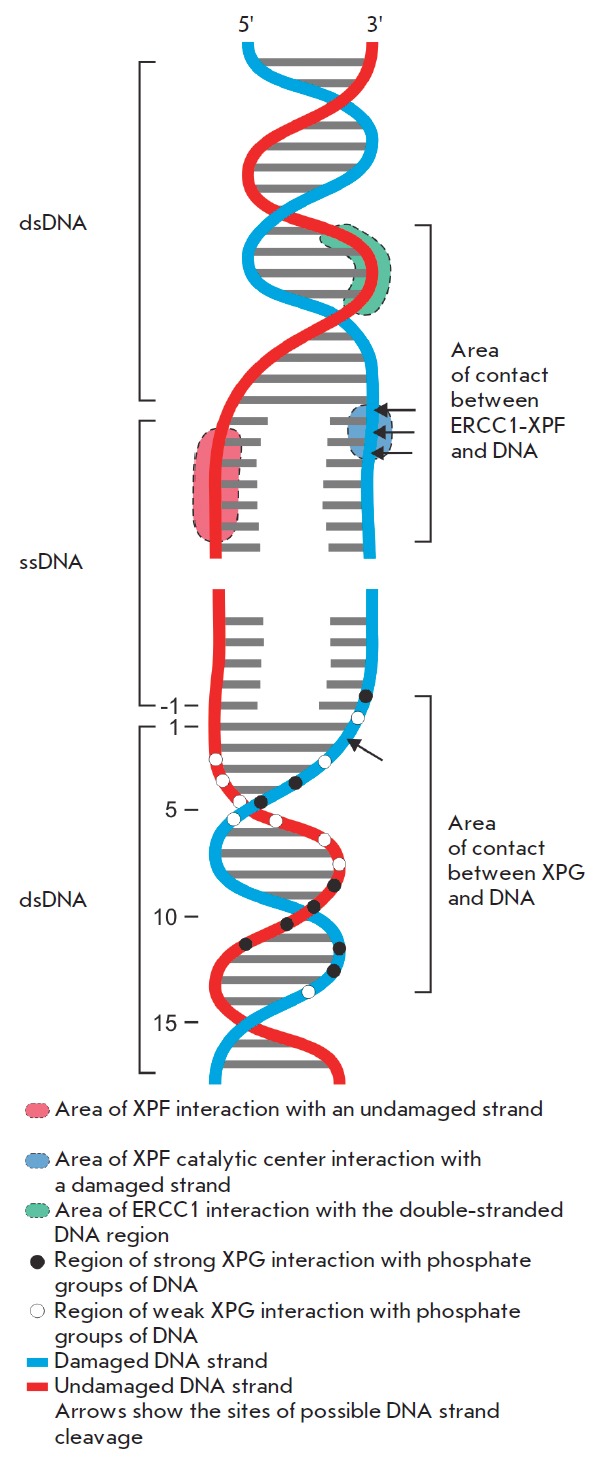
Schematic representation of the XPF-ERCC1 and XPG contacts with DNA in the damage-containing region

The XPG factor acting as a 3'-endonuclease during repair is recruited to a damaged region independently of XPA and RPA, through its interaction with TFIIH [74-76]. XPG-DNA binding and simultaneous release of XPC are the final stage of formation of the complex ready for excision on the DNA. At this step, XPG performs a structural function by stabilizing the open complex; it exhibits no endonuclease activity. ssDNA/dsD-NA transition on the 3’ side of the damage determines the type of XPG interaction with DNA substrates during NER . Various footprinting and gel-retardation techniques show that XPG, together with other members of the flap-endonuclease-1 family, interacts with the double-stranded region of model structures through non-specific contacts with the phosphodiester backbone (*Fig. 3*). These contacts encompass approximately 12 nucleotides of both strands and are located on the external side of the B-DNA helix. The additional nonspecific XPG contacts in single-stranded fragments of model substrates (three contacts with the phosphodiester backbone in a damaged strand and contacts of unknown type with an undamaged strand) poorly affect the binding. At that, the presence of a single-stranded fragment of a damaged strand near the protein binding site is a prerequisite of the demonstration of XPG endonuclease activity [77, 78].

Factor XPF is a structure-specific endonuclease that catalyses incision of DNA at the site of the ssDNA / dsDNA junction on the 5’ side of the damage and functions in NER within a heterodimer with the ERCC 1 protein. An obligate ERCC 1-XPF heterodimer is involved into the complex through the ERCC 1-XPA interaction and breaks the damaged strand on the 5’ side of the damaged site. Identified several domains involved in the functioning of ERCC 1-XPF [79-83]. Both subunits contain a helix-hairpin-helix (HhH) motif required for the formation of a heterodimer near the C-ends [84]. An active center of XPF is a conservative nuclease domain adjacent to the HhH domain [79]. The central ERCC 1 domain is structurally homologous with the nuclease XPF domain; however, instead of the active site with acidic residues, a groove, containing the basic and aromatic amino acid residues, exists in this domain. This fragment interacts with XPA, connecting ERCC 1-XPF to other NER machineries [81, 83]. Individual recombinant XPF domains and the data on archaeal XPF proteins demonstrate that these five domains participate in the interaction with DNA [79-81]. Mass spectrometry, NMR spectroscopy, and *in vitro *analysis of the protein- DNA binding allowed to determine the structure of the complex of the C-terminal HhH domain of the XPF protein with ssDNA in a solution [78]. A stable complex with ssDNA forms an HhH homodimer. At that, DNA is twisted around a protein in a way providing protein- DNA interaction along the phosphate backbone of a molecule. A positively charged fragment in the second helix of one of the HhH motifs comes into contact with the phosphate backbone of ssDNA. These data, along with data in a previous publication [85], allow to construct a model of the ERCC 1-XPF complex. This model explains the positioning of endonuclease at the site of the ssDNA /dsDNA junction on the 5’ side of the damage. According to the model, the ERCC 1 HhH domain interacts with a double-stranded portion of DNA. The nuclease domain of XPF comes into contact with the damaged DNA strand, while the XPF and ERCC 1 HhH domains come into contact with the undamaged strand (*Fig. 3*).

The role of the C-terminal DNA-binding domains in the interaction between heterodimer and DNA substrates was examined through a mutation analysis within full-size ERCC 1-XPF. Mutations in one domain considerably reduced the activity of the NER pathway neither *in vitro *nor *in vivo*. Functioning of the NER pathway is disturbed when mutations are inserted into several domains, and the significance of separate domains is hierarchic [84]. In the presence of catalytically inactive XPG, ERCC 1-XPF catalyzes 5’-incision (15–25 nucleotides away from the damage) and forms an unbound 3’-hydroxyl group required for the initiation of the repair synthesis and emergence of the mobile single-stranded fragment containing the damage. The changes in the structure of the protein-nucleic complex allow an XPG to exhibit catalytic activity [78]. 3’-incision of DNA (3–9 nucleotides from damage) completes the process of damaged site excision. In the structure of XPG, after excision while remaining bound to the DNA, there are motifs that provide specific interaction with PCN A (nuclear antigen of proliferative cells) for some time after excision. XPG might facilitate efficiency and processivity in the repair synthesis [1, 2].

## REPAIR SYNTHESIS

Repair synthesis and DNA strand ligation are performed by the enzymes and protein factors that also participate in DNA replication. The DNA polymerase δ or ε and factors RFC, PCN A, and RPA are needed for DNA synthesis. An RFC complex consisting of five different subunits facilitates ATP-dependent PCN A loading onto DNA near the 3’-end of the DNA fragment flanking a gap resulting from excision. PCN A is a homotrimeric complex that forms a ring-shaped structure sliding along DNA and interacting with DNA polymerases, thus facilitating the processivity of the enzymes [1].

## CONCLUSIONS

A NER process is controlled by multiple weak interactions between proteins and DNA substrates, along with protein-protein interactions in nucleoprotein complexes. In a eukaryotic cell after stable XPC/DNA complex formation during the initial recognition of the damage, NER is actually performed by reparasome, a complex of variable composition and architecture consisting of a large number of subunits. Individual subunits of the complex have no sufficient affinity and selectivity to the substrate (DNA containing bulky damage). The situation changes when specific protein complexes are established at the damage site. The NER proteins of these complexes are joined by the DNA processing. A total of 18 polypeptides must be accurately positioned within two or three DNA turns when a stable structure ready for damage removal is formed and excision starts. The structure of NER -associated proteins provides the possibility of contact with the DNA substrate and of dynamic specific protein-protein interactions. The changes in interactions performed by the same protein are one of the mechanisms that regulate the repair process and fine-tune the complexes, providing high-precision nucleotide excision repair. The study of the composition and architecture of nucleoprotein NER complexes both *in vitro *and *in vivo* requires the use of a broad range of methods and model systems of different complexity.
